# Modeling the impact of future rainfall changes on the effectiveness of urban stormwater control measures

**DOI:** 10.1038/s41598-024-53611-1

**Published:** 2024-02-19

**Authors:** Tyler G. Nodine, Gary Conley, Catherine A. Riihimaki, Craig Holland, Nicole G. Beck

**Affiliations:** 1 2NDNATURE, 500 Seabright Avenue, Santa Cruz, CA 95062 USA; 2 The Nature Conservancy, 322 8th Avenue, New York, NY 10001 USA

**Keywords:** Environmental sciences, Hydrology, Climate-change impacts, Hydrology

## Abstract

The convergence of urban expansion, deteriorating infrastructure, and a changing climate will escalate the risks of stormwater pollution and urban flooding in the coming decades. Using outputs from an ensemble of global climate models to drive a high spatial resolution stormwater model, we analyzed climate change impacts on urban stormwater runoff and control measures for 23 cities across the United States. Runoff model outputs for two future emissions scenarios ending in 2055 were compared against a historical scenario to assess changes. All cities showed increases in average annual stormwater runoff, with changes up to 30% over the next 30 years due to a greater frequency of high intensity storm events. Runoff model outputs showed substantial variation across cities with untreated stormwater runoff increasing by as much as 48%. Patterns of future runoff impacts within cities will affect the performance of distributed treatment strategies such as Green Stormwater Infrastructure (GSI) to meet municipal water quality improvement and runoff reduction goals. Results indicate that adoption of adaptable design standards and decision support tools that readily accommodate projected precipitation changes are critical for supporting more resilient designs of stormwater control measures.

## Introduction

Both model projections^1–3^ and observational data^4,5^ show that atmospheric changes resulting from elevated CO_2_ levels^6^ will bring more frequent high-intensity precipitation events to many parts of the United States. Shifts in precipitation may degrade the future performance of urban stormwater control measures designed to reduce pollution and mitigate local flooding, because local and state design specifications are based on historic, not future, precipitation patterns^7,8^. Expected hydrologic changes include greater stormwater runoff volumes, higher peak flow levels, and increased frequency of events that exceed the capacity of stormwater systems^9–12^. Coupled with the increased surface runoff and flashier hydrologic responses associated with urban development^13,14^, these hydrologic changes may lead to more frequent urban flooding, increased infrastructure stress, and elevated pollutant loading to waterbodies^15–18^. Water pollution ranks as one of the top five threats to aquatic ecosystems and species globally^19,20^ with urban stormwater runoff being a primary source of impacts^21^. If stormwater infrastructure shortfalls are not addressed, climate change will continue to exacerbate impacts to human health, property, and natural habitats. Indeed, studies show insufficient mitigation of runoff impacts in growing cities with aging stormwater systems across the United States^22,23^.

Ensuring that the next generation of stormwater control measures (SCMs), including nature-based strategies such as green stormwater infrastructure (GSI), delivers the expected benefits requires re-evaluation of design standards to explicitly incorporate future climatic conditions. In the United States, regulations under the Clean Water Act require implementation of SCMs with local jurisdictions often requiring GSI to reduce pollution from urban stormwater runoff (e.g.^24^). Climate-related runoff benefits of distributed SCMs, such as GSI, include reduction of local flooding risks^25–27^, recharging groundwater^28,29^, and modulation of flashy runoff responses^30,31^. In addition, GSI can provide habitat protection, air quality improvements, urban cooling, carbon dioxide uptake, and improve the social well-being of communities^32,33^. State regulatory authorities typically set design standards for GSI and other SCMs with the identification of a target design storm^34^, either via precipitation event recurrence (return interval) or by an annual probability of occurrence based on historical precipitation records^35^, such as retaining the stormwater volume produced from an 85th percentile, 24-h storm event^34^. The precipitation data that inform design standards are updated infrequently, and most cities rely on outdated precipitation frequency analyses such as the National Oceanic and Atmospheric Administration’s Atlas 14 reports, last updated in 1973 for several US states^36^. With the average life expectancy of SCMs estimated at greater than 30 years^37^, those already in the ground or currently being planned may be under-designed for near-future climate^7,35^. Regionally variable effects of climate change on the performance of GSI^38^ are likely to contribute to their performance uncertainty which is a key barrier to mainstream adoption of these practices^39^.

Recent reviews of the literature make it clear that substantial uncertainty remains for understanding the impacts of climate change on the effectiveness of GSI^40^ and SCMs in general^41^, along with the need to refine simulation models accordingly. As demonstrated by Wang et al.^40^, there are relatively few examples of studies that focus on the climate change implications for the design of SCMs and impacts on stormwater runoff (e.g.^42–45^). Instead, studies have predominantly focused on the problem of downscaling GCM (global climate model) projections for use in urban drainages^46–48^ and on the effectiveness of conveyance networks to mitigate local flooding under climate change^49,50^. The approach to investigating climate change impacts on stormwater and SCMs has generally been to apply a continuous simulation model over a small study watershed, forced by downscaled projections from a GCM, with outputs lumped at the drainage scale^51–53^, with some studies focusing explicitly on GSI practices^38^. Several studies indicate that projected increases of precipitation intensity may result in reduced efficacy of SCMs given current design standards^41,42,52^.

Key limitations of current modeling approaches for incorporating climate change projections to stormwater planning include sparse data for model calibration at urban-drainage scales, requirement of sub-daily precipitation inputs, and lack of ability to discern sub-watershed response patterns. Given the widespread need to use modeling tools to optimize SCM implementation and adaptation strategies^54^, these technical gaps constrain stormwater managers’ ability to formulate the most effective water quality protection strategies that incorporate future climate changes^55^. Since stormwater management decisions are often made at the scale of individual parcels, models that operate at higher spatial resolution may provide more useful outputs for planning purposes^56^, particularly since the locations and spatial distribution of SCM practices can be a key factor in determining watershed-scale effectiveness^30,31,57,58^. While some studies have employed runoff climate sensitivity factors to discretize lumped model outputs^10^, a more direct approach is to incorporate spatial variation of factors contributing to sub-watershed scale runoff responses directly into the runoff model. This allows the model to be run at high spatial resolution in many watersheds or cities simultaneously. Similarly, the uncertainty incurred with GCM output temporal downscaling procedures^59^ can be directly addressed via a probabilistic treatment of the GCM outputs to drive stormwater runoff predictions^60^. Thus, with this study we have endeavored to fill a key stormwater management data gap: high spatial resolution, broad geographic coverage, planning-level estimates of runoff changes associated with future climate conditions.

**Figure 1:**
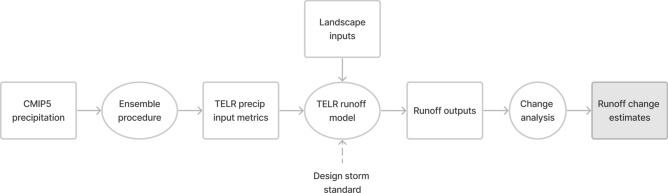
Chart showing the simplified flow of information between input data, the TELR model, and runoff results. Rectangles represent data inputs and outputs and ovals represent calculation steps. See Methods section for full descriptions of data inputs and analysis procedure.

In this study, we used precipitation projections from an ensemble of GCMs included in the Coupled Model Intercomparison Project Phase 5 (CMIP5) to drive a spatially distributed stormwater runoff model to estimate future changes in runoff conditions and distributed SCM performance (Fig. 1). To illustrate projected changes, we selected 23 cities located across the United States, encompassing a diversity of sizes, geographic regions, and rainfall regimes (Table 1). The Tool to Estimate Load Reductions (TELR) stormwater model was used to calculate runoff volumes^56,61^. TELR employs fine spatial resolution process representation (30-m grid cells) and a probabilistic treatment of 24-h event precipitation depths to estimate runoff response patterns (see Methods for a detailed model description). Daily precipitation data from the CMIP5 climate models for two future emissions scenarios (RCP 4.5 & RCP 8.5 for the period 2025–2055) and a historic scenario (1975–2005) were processed and ensembled for use as inputs to the runoff model. After validation of the runoff model using long-term gauge data from the US Geological Survey (USGS), for each study city we estimated total runoff and potential runoff captured by distributed SCMs given a common design storm standard and projected climate conditions. Runoff model outputs for the two future emissions scenarios were compared against the historical scenario to estimate percent changes in runoff and distributed SCM performance. Through interpretation of these results, we explore how the integration of climate change projections with high spatial resolution stormwater modeling tools can inform more resilient stormwater control implementation strategies.

**Table 1:** Study cities organized by region with key descriptive characteristics.

City	State	Region^a^	Region number^a^	Area (km^2^)^b^	Population^b^	Climate class^c^	Mean annual PPT (cm)^d^
Albany	NY	Northeast	2	56.7	100,742	Humid	101.9
Philadelphia	PA	Mid-Atlantic	3	364.7	1,587,761	Humid	121.7
Washington DC		Mid-Atlantic	3	161.4	674,875	Sub-humid	112.3
Nashville	TN	Southeast	4	1286.7	672,371	Humid	130.6
Charlotte	NC	Southeast	4	776.2	838,742	Sub-humid	114.0
Atlanta	GA	Southeast	4	347.1	464,043	Humid	130.6
Tampa	FL	Southeast	4	305.9	370,224	Sub-humid	126.2
Milwaukee	WI	Great Lakes	5	250.7	591,865	Sub-humid	86.9
Chicago	IL	Great Lakes	5	598.3	2,781,116	Sub-humid	99.1
Columbus	OH	Great Lakes	5	577.8	871,273	Sub-humid	102.9
Albuquerque	NM	South Central	6	490.8	567,516	Arid	26.7
Austin	TX	South Central	6	790.2	935,806	Semi-arid	86.6
Lincoln	NE	Midwest	7	234.1	283,989	Sub-humid	79.5
St. Louis	MO	Midwest	7	170.9	316,262	Sub-humid	108.2
Billings	MT	Mountains and Plains	8	112.7	115,030	Semi-arid	35.6
Denver	CO	Mountains and Plains	8	400.4	699,521	Semi-arid	41.7
Salt Lake City	UT	Mountains and Plains	8	289.3	201,705	Semi-arid	49.8
Las Vegas	NV	Pacific Southwest	9	614.6	642,798	Arid	12.2
Phoenix	AZ	Pacific Southwest	9	1341.4	1,601,381	Arid	22.9
Salinas	CA	Pacific Southwest	9	60.1	155,320	Semi-arid	37.6
Los Angeles	CA	Pacific Southwest	9	1225.1	3,986,442	Arid	40.6
Seattle	WA	Pacific Northwest	10	258.0	687,870	Humid	98.3
Portland	OR	Pacific Northwest	10	375.8	637,683	Humid	112.3

^a^US Environmental Protection Agency (EPA) regions (https://www.epa.gov/aboutepa/regional-and-geographic-offices).

^b^2020 US Census^62^.

^c^Aridity index^63^.

^d^PRISM^64^.

## Results

### Regional patterns of precipitation and runoff changes

**Figure 2:**
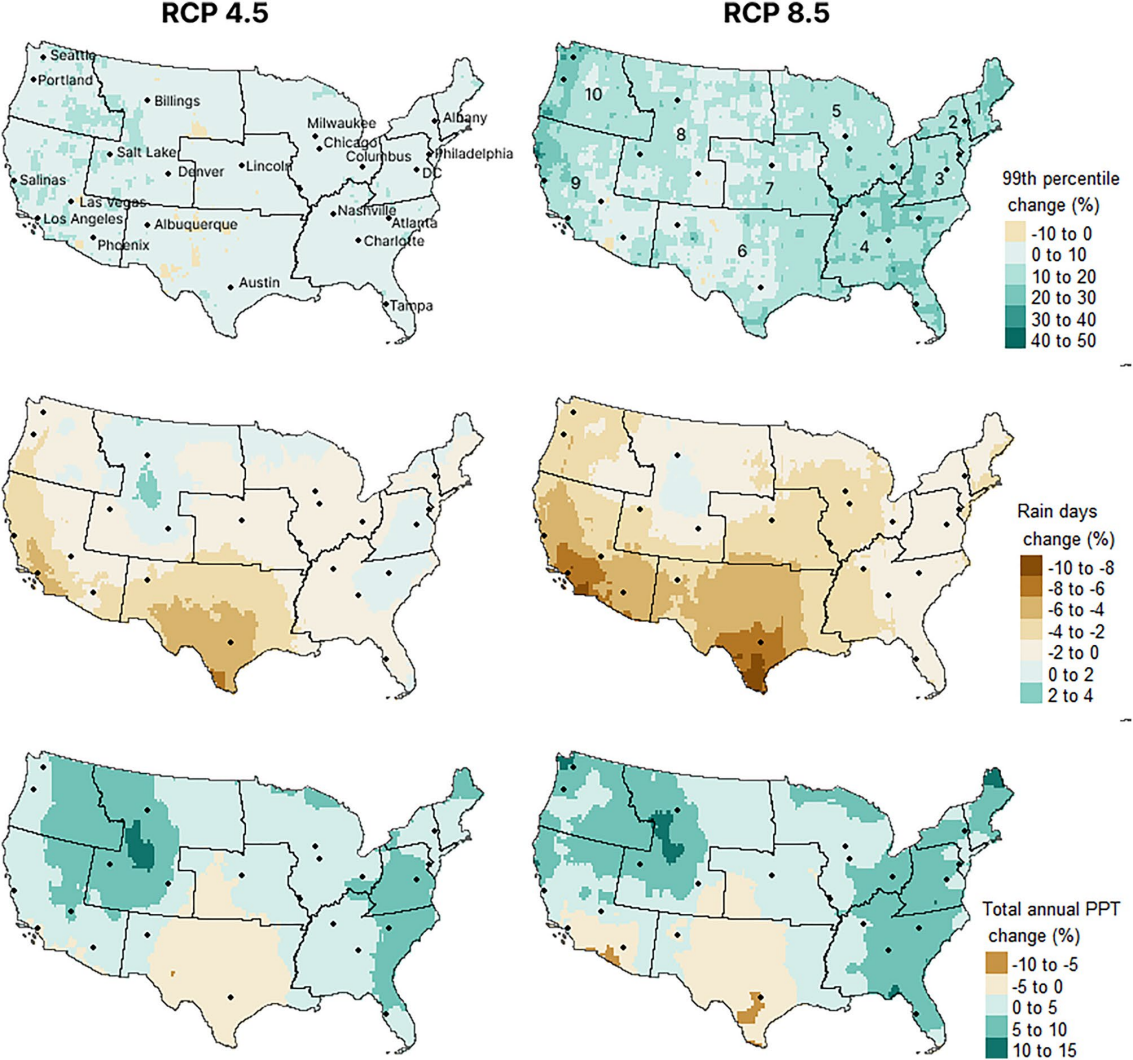
Projected precipitation changes for RCP 4.5 and 8.5 for 2025–2055 relative to the historic reference period (1975–2005). Regional boundaries and numbers displayed are derived from US Environmental Protection Agency (EPA) regions. Maps were generated using Rstudio version 1.3.1093 (https://posit.co/products/open-source/rstudio/) (R Core Team 2021).

All precipitation metrics calculated from the GCM ensemble showed sensitivity to projected climate in future scenarios (2025–2050) relative to the historic period (1975–2005) (Fig. 2). Ensemble outputs for the future period indicate increases in extreme precipitation events, characterized by the 99^th^ percentile event, across nearly all the conterminous United States for both emissions scenarios analyzed. Under RCP 4.5, described by the IPCC as the ‘moderate’ emissions scenario, increases in 99^th^ percentile events ranged from 0 to 20% for most of the country, while increases under RCP 8.5 are generally larger—up to 50%. In contrast, the number of annual rain days is estimated to decrease for most parts of the United States, though more modestly for both scenarios, with a range of -10 to + 4%. Total annual precipitation delivered depends on changes to both event magnitudes and the number of rain days. Total annual precipitation is projected to decrease in the Southwest while increasing along the East Coast and in the Pacific Northwest (Fig. 2). The spatial and temporal patterns observed in these outputs align closely with the expected changes outlined in the IPCC Fifth Assessment report^2^ and the 4th National Climate Assessment^3^.

Historic data and the projected precipitation data were used to drive the TELR model and calculate differences between the historic and future scenarios. In validation testing, TELR showed strong correspondence with gauge data in 219 urbanized watersheds across the US during the historic period, both in terms of random errors (R^2^ = 0.83) and systematic offset (PBIAS = −7.1). These results provide confidence that the model was able to capture the dominant long-term precipitation-runoff response modes across a wide variety of watersheds.

**Figure 3:**
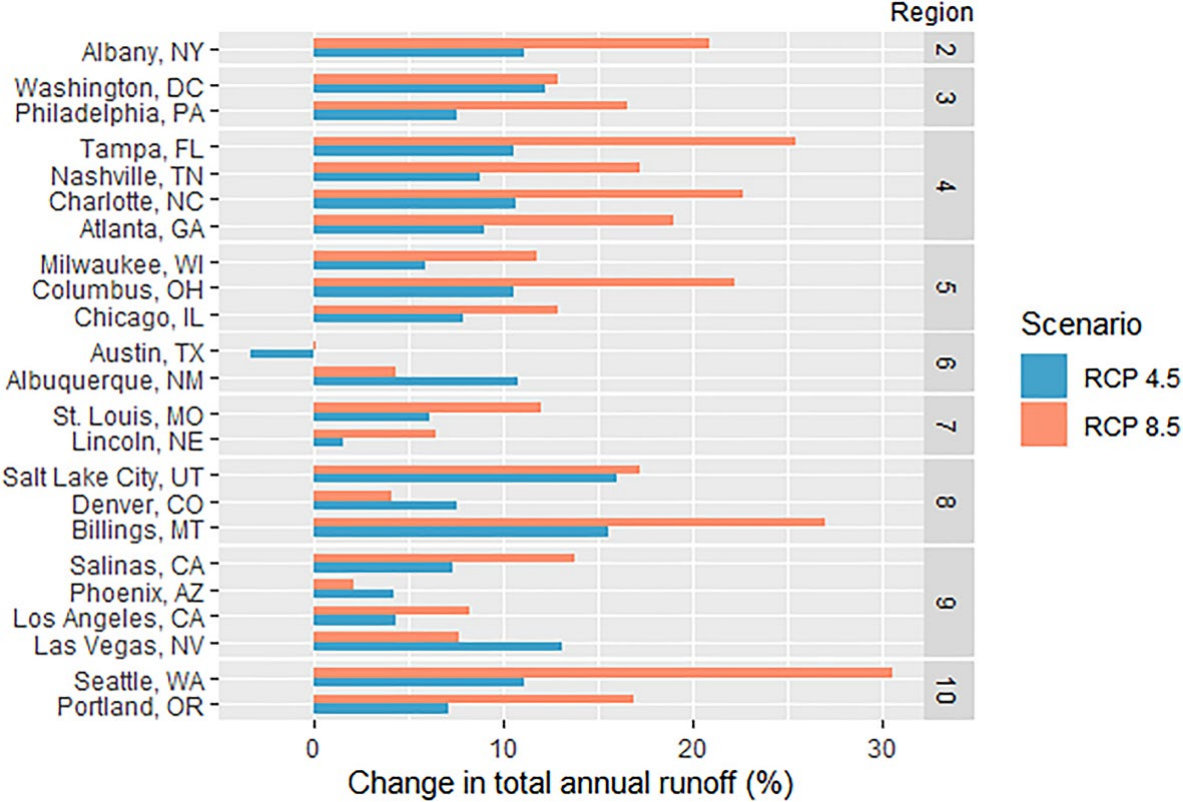
Change in total annual runoff by city for each RCP scenario for 2025–2055 relative to the historic reference period (1975–2005).

Runoff modeling results for the 23 study cities shows that under both RCP scenarios annual stormwater volumes are expected to increase for nearly all study cities. Changes across all study cities ranged from -3 to 16% for RCP 4.5 and 0 to 31% for RCP 8.5 (Fig. 3). The magnitude of runoff increases under RCP 4.5 show lower variance across cities compared to the increases under RCP 8.5. In the RCP 8.5 scenario, moderate increases are estimated for the southwestern United States (i.e., Albuquerque, NM; Phoenix, AZ: Las Vegas, NV; Los Angeles, CA), while larger increases are expected for most cities along the East Coast and in the Pacific Northwest, with Seattle, WA, showing the largest increase. Austin, TX was the only United States city that showed a decrease in total annual runoff (-3% under RCP 4.5) under future climate conditions, due to decreases in annual rain days and mild changes in extreme event depths projected by the GCM ensemble.

**Figure 4:**
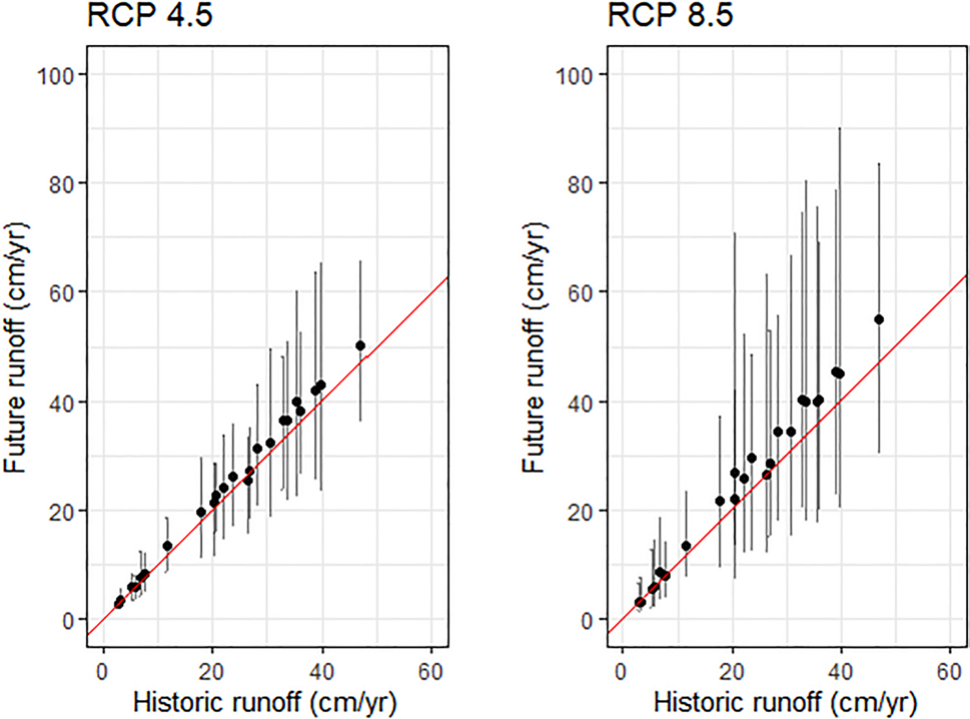
Comparison of total annual runoff estimates for historic (1975–2005) and future (2025–2055) climate scenarios. Black dots indicate the GCM ensemble mean and error bars represent runoff estimated from the GCM ensemble’s 25th and 75th percentiles. The 1:1 line is shown in red.

Some of the variability in runoff changes across cities must be attributed to the GCMs themselves. RCP 8.5, which represents atmospheric CO_2_ concentrations that deviate further from observed conditions, shows a greater lack of precision across models in future precipitation projections than RCP 4.5 (Fig. 4). According to outputs driven by some individual GCMs, future annual runoff in these cities could be much larger than the ensemble mean suggests. While some cities show wide ranging runoff changes across GCMs, particularly under RCP 8.5, most models agree on the direction of change. For several cities, the range of predictions across models is larger than the expected difference from past to future runoff. Ensembling helps to make the best use of all model outputs since it is impossible to know which model may provide the ‘most correct’ representation of future conditions.

**Figure 5:**
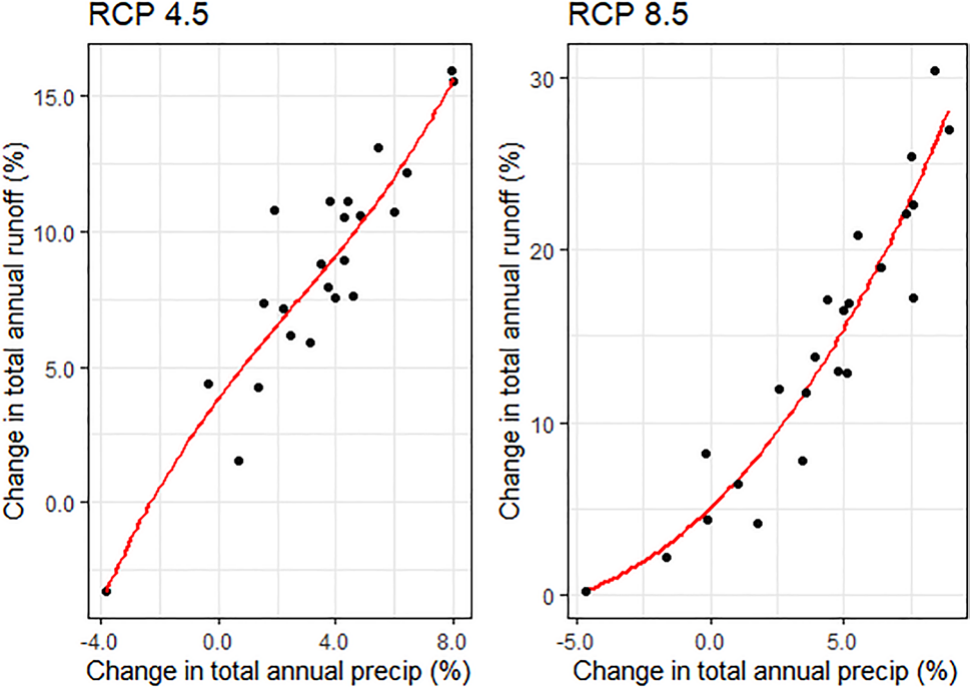
Changes in total annual precipitation and total annual runoff for each study city under RCP 8.5 compared to the reference period (1975–2005). A polynomial spline interpolation is shown in red.

For a given increase in annual precipitation, we see a proportionally greater increase in runoff for both RCP 4.5 and 8.5 (Fig. 5). This largely reflects the impact of more extreme events, with more intense precipitation delivered during fewer rain days, which produces more runoff per unit of precipitation compared to smaller events. As such, total annual runoff is estimated to increase even for those cities which see an overall decrease in total annual precipitation. Gaps between precipitation and runoff changes are also influenced by levels of impervious cover and soil permeability; both of which are inputs to the TELR runoff model. Areas or cities with high impervious cover and low soil permeability show greater increases in runoff volumes from projected increases in rainfall event intensity.

**Figure 6:**
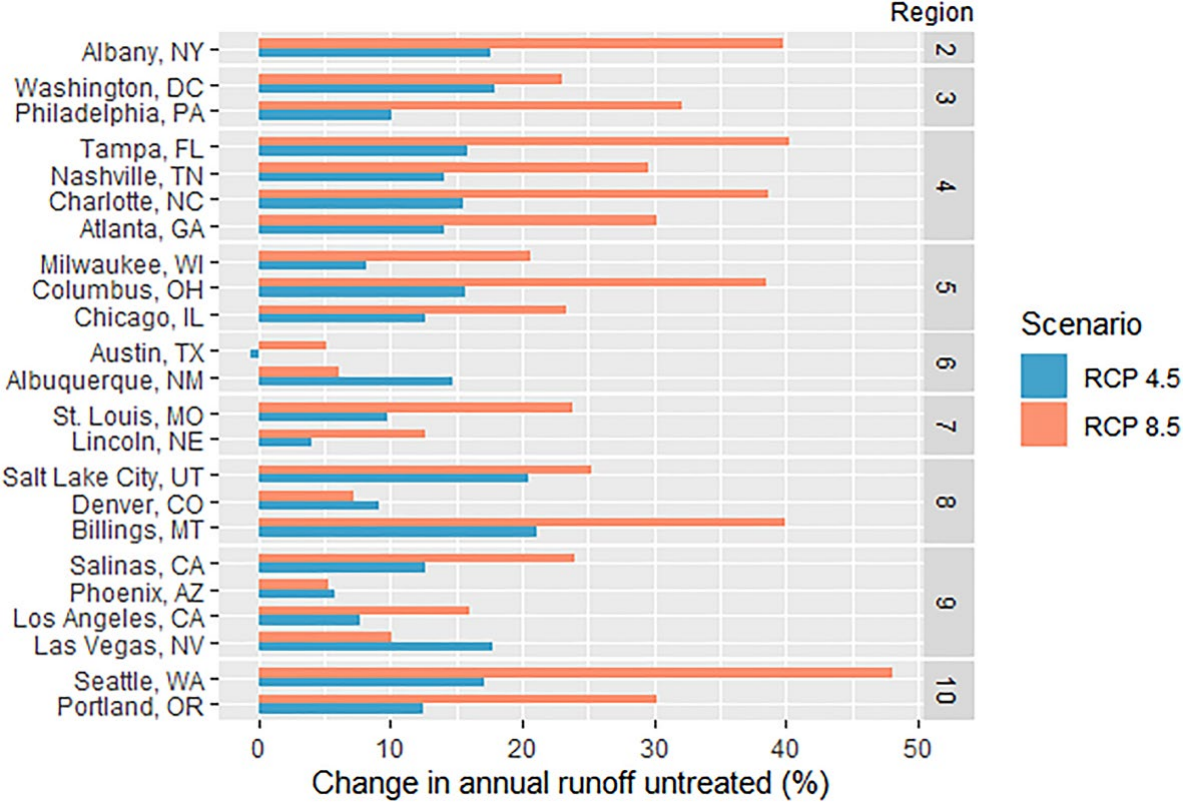
Percent change in untreated runoff, assuming design standards remain at the historic 85th percentile.

Given current design standards, untreated runoff resulting from flows that exceed SCM capacities is estimated to increase in nearly all cities, with a range of -1 to 21% for RCP 4.5 and 5 to 48% for RCP 8.5 (Fig. 6). Untreated runoff volumes were calculated as runoff from events above the historic 85th percentile 24-h depth, a commonly used stormwater control measure design standard in cities throughout the US. Percent changes in bypassed flows resulting from rainfall depths exceeding the historic 85th percentile depth were greater than changes to total runoff volumes (see Fig. 3). This reinforces a key feature of expected runoff increases: they will be driven by larger extreme events, rather than more frequent smaller events. The differences in untreated runoff across cities (Fig. 6) primarily reflect a combination of (1) changes in the occurrence probability of different event depths, (2) changes to the number of rain days that occur each year, and (3) interaction of these rainfall variables with landscape factors that affect runoff generation. Only Austin, TX showed a decrease in this metric and only under the RCP 4.5 scenario. These results indicate that SCMs that employ the 85th percentile standard informed by historic precipitation records in cities such as Austin, TX and Phoenix, AZ may be adequately designed to handle future climate changes, while SCMs in cities like Billings, MT and Seattle, WA may be significantly undersized and unable to provide their current levels of water quality benefits in the future.

### Intra-city patterns of runoff change

**Figure 7:**
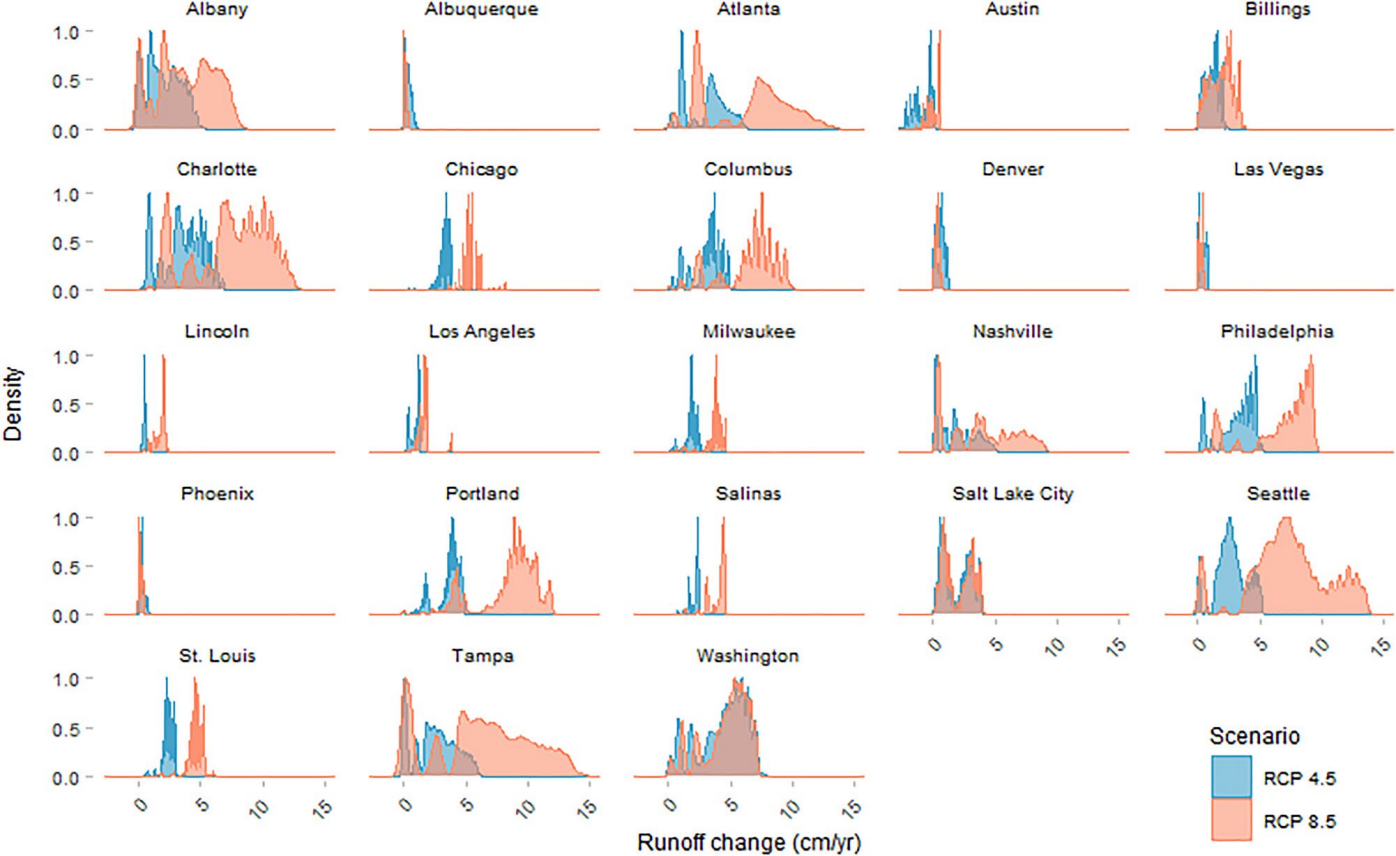
Density histograms of total annual runoff changes within cities by climate scenario, representing the proportion of grid cells within each city boundary.

The study cities showed distinct patterns of runoff increases across their areas and between the two emissions scenarios. Density distributions are shown in Fig. 7, with values representing the proportional area within each city estimated to experience a given runoff depth change under the projected climate scenarios. As we would expect, runoff increases under RCP 8.5 generally show a positive shift and a wider spread relative to RCP 4.5. The exceptions to this pattern are cities in drier regions (e.g., Albuquerque, NM; Las Vegas, NV; Phoenix, AZ; Denver, CO), which show more moderate increases across city areas (Fig. 7). The magnitude of the positive shift varies by city, indicating that some cities may have a narrow range of expected future runoff changes, while in other cities, patterns of runoff changes may vary significantly depending on the carbon emissions trajectory. In both cases, the estimated runoff pattern changes reflect the combination of the expected shift in the precipitation distribution and city-specific runoff generation factors.

**Figure 8:**
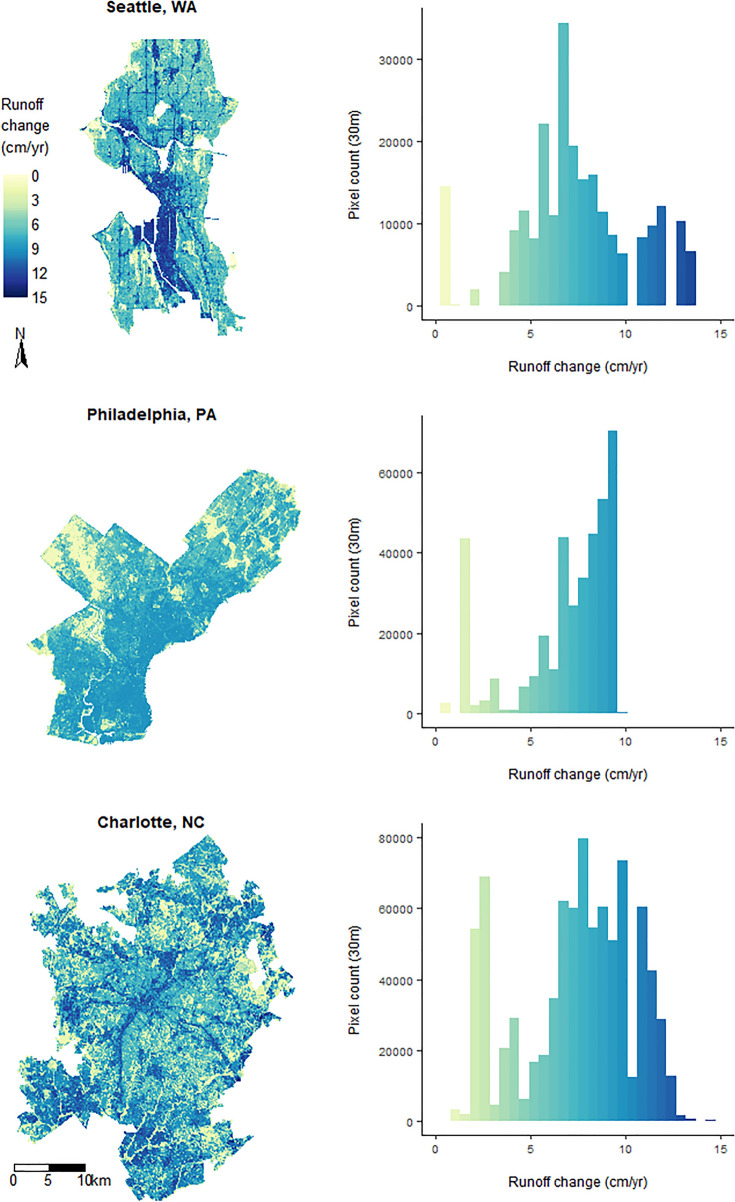
Runoff difference maps and histograms for three study cities, showing the variation of runoff change patterning across and within cities. Maps were generated using Rstudio version 1.3.1093 (https://posit.co/products/open-source/rstudio/) (R Core Team 2021).

Cities also showed considerable differences in how runoff increases were distributed over space. Figure 8 shows runoff increases for three cities under the RCP 8.5 climate scenario and highlights areas estimated to be most at risk in terms of runoff increases. Patterns of runoff change across cities result from the interaction between precipitation pattern shifts and landscape factors. Since the GCM ensemble outputs have a much coarser resolution (28 km) compared to other inputs, which are defined on a 30-m grid (hydrologic soil properties, impervious cover, and land use), the observed patterns within specific cities largely reflect the hydrologically unique combinations of those landscape factors. The greatest increases tend to be along roadways and in intensively developed parts of the cities, indicating that the areas with high impervious cover will be responsible for the largest increases in runoff under both future climate scenarios. In Seattle, WA, high impact areas are primarily concentrated in the downtown and the industrial district that spreads southward from the shoreline, whereas both Philadelphia, PA and Charlotte, NC have the highest-level increases spread more uniformly across the entire urban area. Of these three cities, Charlotte shows the highest spatial frequency variation of runoff changes across the city, with small pockets of large increases surrounded by areas of lower increases.

## Discussion

Stormwater planning to build local climate change resiliency will require a detailed understanding of how future conditions will affect the design, location, and sizing of SCMs. Research has shown that SCMs can play a critical role in mitigating runoff increases associated with climate change^51,52^. Our results indicate that the amount of runoff generated annually could increase up to 30% for many cities in the United States by 2050, with projected precipitation to be delivered in fewer, more extreme precipitation events, driving disproportionate runoff increases. The critical implication is that SCMs in much of the United States are likely undersized to achieve planned water quality benefits or mitigate local flooding risks in the future since SCMs are expected to exceed their capacities more frequently than in the past. The local increase in runoff in cities like Seattle, WA, Billings, MT, Columbus, OH, Tampa, FL, and Albany, NY differs between RCP 4.5 and RCP 8.5, indicating that stormwater runoff volumes will be sensitive to the exact climate pathway experienced. Our results underscore the importance of developing the technical tools that will allow local jurisdictions to readily integrate climate change projections into local planning processes and modeling tools. Current availability of CMIP ensembles within Google Earth Engine^66^ is an example of a step in that direction. Other helpful advancements going forward would include a national standardization of GCM projections and runoff modeling integration, including climate model ensembling procedures, uncertainty quantification, and selection of emissions scenarios with defined time horizons.

As noted by prior researchers, relatively few modeling studies are available that focus on urban stormwater management in the context of climate change^67^, and since climate impacts vary substantially by location, identifying variation across cities is imperative^42^. The work available corroborates this study’s findings that in regions likely to experience precipitation regime shifts, runoff increases will be acute in urbanized areas due to both precipitation intensity changes and continued urban expansion^68,69^. For example, Zahmatkesh et al.^53^ used statistical downscaling of the CMIP5 outputs and a change factor approach to drive a continuous simulation model that showed an average of 51% increase in total annual runoff across New York City for the future period 2030–2059. Blair and Sanger^70^ applied the Stormwater Runoff Modeling System (SWARM) to quantify changes in stormwater runoff relative to the 95th percentile 24-h storm event, with runoff volume increases of 64% to 90% for the moderate scenario (10% rainfall increase) and from 158 to 243% for the severe scenario (20% rainfall increase). Modeling using the Soil Water Assessment Tool (SWAT) by Kiprotich et al.^71^ showed that runoff changes in Nairobi, Keya projected to 2089 ranged from -13% (RCP 4.5) to 77% (RCP 8.5) and 29% (RCP 4.5) to 179% (RCP 8.5) when future development was considered. While the studies cited examined urban runoff response changes for individual cities, our work provides a way to examine such changes within a common framework across a wider geographic scope at high spatial granularity.

The results of our modeling point towards the need for increasing SCM capacity and adapting their locations and configurations in response to urban stormwater runoff increases, which corresponds with other recent work on this topic. Zhang et al.^44^ used an empirical downscaling to quantify runoff changes in a future period (2040–2049) in Melbourne, Australia to illustrate that future climate will reduce the reliability of water quality treatment by GSI and recommended larger capacity designs to mitigate that uncertainty. Pyke et al.^51^ used a simple stormwater model to quantify the sensitivity of runoff impacts to future precipitation intensities and the benefits of Low Impact Development practices in areas with high impervious cover. Sarkar et al.^38^ used the Regional Hydro-Ecologic Simulation System (RHESSys) to quantify reduced future performance of GSI under a range of mid-century climate change scenarios. Through a systematic meta-analysis of the literature through 2017, Sohn^72^ concluded that the ability of GSI to reduce runoff volumes is expected to decrease with climate-driven increases in storm intensity and frequency. The current study has endeavored to fill the persistent research gaps highlighted by Sohn^72^, which include the need for tools to facilitate site-scale planning of GSI and optimizing the spatial arrangement of GSI for effective stormwater management under future rainfall regimes.

Without policies and regulations to drive more resilient stormwater infrastructure design, runoff increases and SCM capacity shortfalls will lead to greater environmental and social impacts associated with increasing pollution and local flooding risks. Impacts may include impaired aquatic ecosystems, property damage, and human health risks, which will depend on several locally defined factors in addition to runoff increases. In many cities, impacts will be further exacerbated by the continued expansion of impervious cover^68,73,74^ and sea level rise in coastal communities. With stormwater infrastructure reaching the end of its useful lifespan in many parts of the United States and the potential for new funding sources to assist cities with upgrades^23,75^, many cities may soon be able to increase investments to minimize impacts. Further analysis of our results showed that the average increase in treatment capacity required to accommodate projected runoff changes was 21%, which will increase capital costs for their construction accordingly. Such investment decisions also provide an opportunity to take into account the environmental justice implications of the disproportionate impact that projected climate change impacts are likely to have on socially underserved communities^76^.

Coupled with design standards informed by climate change projections, modern stormwater strategies such as GSI can build climate resilience by delivering multiple types of benefits to communities^51^. In addition to capturing, slowing, and treating urban runoff, GSI can mitigate climate change impacts such as rising temperatures and heat island effects by increasing vegetation cover^77^. To date, GSI effectiveness at watershed scales has been hampered by shortfalls in appropriate climate-specific design^54^, implementation density^78,79^, and optimal spatial arrangement^57,58^. As the integration of GSI to stormwater planning gains traction, it is increasingly important for municipal stormwater managers to have the appropriate decision-support tools available to assess cost and benefit trade-offs^78,80,81^, mitigate uncertainty, and maintain flexibility associated with different implementation options^82^. Several of the current gaps can be ameliorated with use of models that allow discernment of spatially precise runoff patterns in response to projected precipitation changes (such as in Fig. 8), providing outputs that are readily interpretable to stormwater managers, city engineers, and urban planners.

Improving output uncertainty derived from various sources will give stakeholders more confidence and clarity in utilizing future runoff projections. GCMs included in the CMIP5 collection have different structures, process representation, and parametrization assumptions that lead to different outcomes^83^. Some of these differences were reflected in the spread of the interquartile range of models within the ensembled runoff estimates of our results (see Fig. 4). Indeed, analyses have shown that the GCM selection procedure can have a greater impact on hydrologic modeling results than the selection and parameterization of runoff models themselves^84^ and increasingly authors are calling for more careful model selection and ensembling procedures^59,85^. Similar to the current study, in their ensemble of 8 GCMs, Zhang et al.^44^ found that high variability across GCM outputs was a key source of uncertainty in the runoff outputs. While we performed an ad-hoc exploratory analysis of individual model performance (see Methods), a comprehensive sensitivity analysis to address the impact of inclusion or exclusion of specific models and different ensembling procedures was beyond the scope of this research. Instead, we relied upon a standard approach to using the full ensemble of CMIP5 GCMs available, which aligned well with the historically observed precipitation from PRISM. Although the stated purpose of the GCM projections used in this analysis is to explore changes at the scale of towns and cities^86^, their coarse spatial resolution (28 km) means that spatial patterns of precipitation change are represented with less precision than that of the other runoff model inputs. Finally, spatial patterns of future runoff impacts will surely be influenced to some degree by urban development. While such changes would largely be reflected in BMP design standards for specific drainage areas, coupling the TELR model with outputs from a land use change model would allow a specific accounting for shifting patterns of impervious cover associated with that development.

## Conclusion

Ensembled climate change projections combined with urban runoff modeling suggest stormwater runoff volumes will substantially increase in many cities across the United States. If design standards for stormwater control measures (SCMs) continue to rely exclusively on historical precipitation records, projected runoff increases will result in up to 48% more untreated runoff from SCMs in the future, largely due to more frequent occurrence of extreme precipitation events. These changes are likely to contribute to continued degradation of water quality and ecological health of waterways and greater uncertainty associated with the performance of distributed SCMs. Impacts are likely to be felt most strongly in historically underserved communities which typically have higher density development with greater impervious coverage and a lower level of service delivered by municipal infrastructure. To reduce the risks of backsliding on water quality improvement progress, cities will need to invest in building stormwater treatment features designed to cope with future rainfall regimes. Decision support tools that can inform both sizing and strategic implementation of SCMs are needed for robust stormwater planning within the context of changing climate conditions. Such tools must be made more accessible to the practitioners who plan and design municipal stormwater mitigation strategies and who currently operate under a dearth of knowledge on how shifting rainfall patterns will impact their planning decisions. Sustainable stormwater planning going forward will include practical approaches to integrate projected climate to urban runoff models, identifying patterns of expected runoff changes within cities to optimize the locations of stormwater control measures, and using GSI to build climate resilience.

## Methods

### Study cities

In our selection of study cities, we considered the distribution of city population sizes, geography, climate, and regulatory jurisdiction. Aridity index^63^ and historic mean annual precipitation^64^ were used to ensure cities represented a range of climate types. Geographic distribution of the study cities was stratified by Environmental Protection Agency’s (EPA) regional jurisdictions, which typically set SCM design standards in coordination with state regulators. We limited our search to cities with populations greater than 100,000 according to the 2020 US Census^62^. Cities were selected based on size (populations greater than 500,000 population were favored), name recognition, and knowledge of leadership and innovation in the stormwater industry. The resulting 23 study cities represent differing climate and regulatory regions, span 22 US states, and have populations ranging from 100,000 to over 4 million (Table 1). City boundaries used for the analysis were obtained from the 2020 US Census^62^.

### GCM data, time horizons & representative concentration pathways

Global precipitation scenarios derived from 22 GCMs that comprise the Coupled Model Intercomparison Project Phase 5 (CMIP5) were obtained from the NASA Earth Exchange (NEX) Global Daily Downscaled Projections (GDDP) dataset^86,87^. NEX-GDDP outputs were accessed and processed through the Google Earth Engine platform^66^. The GCMs included in CMIP5 supported the Fifth Assessment Report of the Intergovernmental Panel on Climate Change^2^ and are widely used in climate research. The names and developing institutions of each GCM included in the NEX-GDDP dataset are provided in supplementary material Table S1. Bhowmik et al.^88^ and Raju and Kumar^83^ provide details on the performance of individual models for the United States. While use of the CMIP6 results may have been preferable, at the time of this work they were not available within the Google Earth Engine platform, which provides the only viable processing platform given the geographic extent of the study. Precipitation projections are provided in NEX-GDDP for two future greenhouse gas emissions scenarios as well as for a historic (pre-2005) scenario. The NEX-GDDP climate projections are provided from NASA spatially downscaled to 28 km, using the Bias-Correction Spatial Disaggregation method^87,89^.

We used 30-year climate scenarios for historic (October 1, 1975-September 30, 2005) and future (October 1, 2025-September 30, 2055) timeframes to represent long-term climate patterns. The historic 30-year time frame was selected to represent the recent past and correspond with the periods upon which SCM design standards are commonly based. The hindcasted precipitation scenarios from the CMIP5 GCMs go through 2005, which provided the upper limit of our historic period. The future time horizon was selected to represent the near future. While many climate studies focus on changes expected by the end of the twenty-first century, we choose a mid-century time frame to make estimates from this study relevant to the lifespan of stormwater infrastructure currently in the ground or being planned. We incorporated precipitation projections for two future greenhouse gas emissions scenarios known as representative concentration pathways (RCPs), RCP 4.5 and RCP 8.5^90^. RCP 4.5 commonly serves as the ‘moderate’ scenario in which anthropogenic CO_2_ emissions level off until 2050 before decreasing^91^. Conversely, RCP 8.5 represents a pathway in which emissions continue to increase along the current trajectory through 2050. Analyzing two future climate scenarios gives decision makers a better understanding of the range of the possible runoff conditions that may be realized in the future. We focus primarily on relative differences between the historic and future climate scenarios to make comparisons across different climate regimes. This approach also minimizes the impact of errors in the absolute change magnitudes on the interpretation of the results.

### GCM ensembling

Climate scenarios in the NEX-GDDP dataset are provided for 22 CMIP5 GCMs. We explored selecting specific models, ensembling a subset of models, and ensembling the full set of GCMs. GCM ensembling, a common approach in climate modeling^83^, entails statistically summarizing outputs from multiple models, which has the benefit of compensating for errors in individual models. Raju and Kumar^83^ and references cited therein highlight the benefits of using a multi-model ensemble approach including minimization of bias and increase in “skill, reliability, and consistency of model forecasts”.

We tested the performance of the GCM ensembling approach based on alignment of hindcasted projections with historically observed precipitation data from the PRISM Climate Group^64^ for the period 1980–2005. This time frame was adjusted from the historic period used in our experimental analysis to accommodate the 1980 start date of the PRISM dataset. Average total annual precipitation and other statistical metrics required for the runoff modeling were used to test performance on the historic data (Fig. S1). We did not perform a formal sensitivity analysis of the inclusion/exclusion of individual models within the ensemble, but we did observe significant variation in future precipitation projections across individual models. The full ensemble showed consistent agreement with historic data across all precipitation metrics and cities (Fig. S1), which suggested that the mean ensemble approach was able to compensate for individual model biases^83^. Given these observations, we determined a mean GCM ensemble was most suitable for this analysis, which is the same approach used for IPCC assessments^2^ and US National Climate Assessment reports^3^. Runoff model outputs generated from the hindcasted GCM mean ensemble and the PRISM historic data were compared, showing strong alignment with an r-squared value of 0.82 with a percent bias of -14.2% (runoff underestimation using the hindcasted GCM data) (Fig. S2), indicating the TELR model had a similar runoff response with the two input precipitation datasets for the historic period. The multi-model ensemble method also allows for estimating the precision of future runoff predictions^84^, which was represented by the 25th and 75th percentile ranges of model estimates.

Precipitation metrics required for runoff modeling included daily precipitation depth probabilities (12.5th, 50th, 85th, 95th, and 99th percentiles) and a count of average annual days with measurable precipitation above 2.54 mm (0.01 inches) for the 30-year study periods. These metrics were calculated from daily precipitation depths for each CMIP5 GCM model included in the NEX-GDDP dataset. The outputs generated from each model were combined in the final step of precipitation data processing, in which the ensemble mean was calculated for each specific raster grid cell.

### Runoff modeling

To estimate runoff, we used the stormwater Tool to Estimate Load Reductions (TELR) described in Beck et al.^61^, Conley et al.^56^, and Conley et al.^92^. The TELR model employs a hybrid event-based approach that combines a set of 24-h events drawn from long-term precipitation probability distributions to quantify the range of expected runoff responses. The model is spatially distributed, with all calculations performed on a 30-m raster grid. Within TELR, urban runoff from a grid cell R was calculated via a Riemann sum of the flows generated by each 24-h precipitation event up to the xth percentile per Eq. (1):

1 $$R={\int }_{0}^{100}Q\left(x\right)dx\approx \frac{1}{2}{\sum }_{k=1}^{N}\left({x}_{k+1}-{x}_{k}\right)*\left(Q\left({x}_{k+1}\right)+Q\left({x}_{k}\right)\right)*d$$

where Q is the runoff volume for the xth percentile storm (0–100), k is a number in the sequence of total N percentile events used to estimate the integral, and d is and the number of annual rain days. The total runoff volume for a drainage or region of interest is the sum of R for each 30-m grid cell within the geographic area.

Daily precipitation raster layers for the time horizon of interest were used to calculate the precipitation percentile values and average annual days of rain used to drive runoff generation in TELR for each grid cell. Runoff transformation in TELR is accomplished via the Natural Resources Conservation Service (NRCS) curve number (CN) method and the approach detailed in Technical Release 55 (TR-55) to estimate runoff from small urban catchments^93^. The CN defines the fraction of flow that infiltrates over previous surfaces and the fraction of stormflow runoff generated during storms based on hydrologic properties of the soil and land cover over a range of rainfall depths. Readers are directed to the USDA^93^ for additional details on the CN methodology and Beck et al.^61^ for further description of its implementation in TELR.

The CN runoff is parameterized by hydrologic soil type and impervious cover, both of which are available as gridded datasets for the entire US. The NRCS SSURGO database^94^ was the primary data source for soils within city boundaries, and the STATSGO2 database^95^ (which provides coarser resolution) was used to fill in spatial gaps in coverage that occur in the SSURGO data. Each dataset was rasterized and downscaled to 30-m resolution. Impervious cover is specified using 2019 data from the National Land Cover Dataset which is provided at 30-m grid cell resolution^96^.

A key advantage to this approach for climate change applications is that it avoids the additional uncertainty incurred with downscaling daily climate projections to hourly or sub-hourly values as required for continuous simulation runoff models^97,98^, which may constitute the largest source of uncertainty for both climate ensembles^59^ and hydrologic impact projections^99^. Thus, the type of probabilistic approach employed by TELR is particularly amenable to forecasting climate change impacts on hydrological systems due to their efficiency for incorporating multiple models, parameterizations, or ensembling approaches^60,100^. While use of daily inputs by TELR matches with the GCM outputs, and allows much greater coverage at high spatial resolution, it limits the types of runoff changes that can be investigated. Changes that depend strongly on hourly or sub-hourly precipitation dynamics such as peak flow levels would be better suited to a continuous modeling approach, requiring temporal downscaling of climate model inputs and typically performed at much coarser spatial resolution. Given that we are exploring aggregate runoff changes at the 24-h event scale, which also matches the scale of most SCM design standards, the probabilistic framework employed by TELR provides a reasonable approach for linking climate model outputs to runoff impacts^60^.

### Stormwater runoff model performance validation

**Figure 9:**
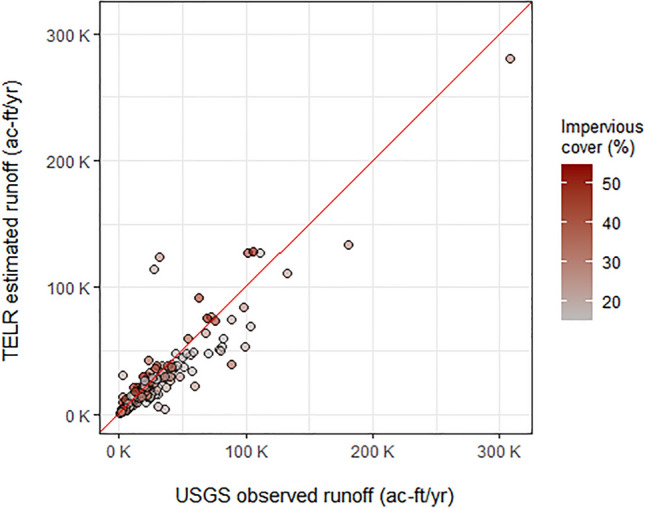
Results of watershed-scale comparisons of TELR modeled and gauged runoff data. The 1:1 line is shown in red and impervious cover indicated with color ramp.

To facilitate validation of the TELR model, we selected a set of watersheds based on U.S. Geological Survey (USGS) streamflow data availability^101^ and proportional impervious coverage as defined by the National Land Cover Database (NLCD)^96^. Potential watersheds were filtered using two criteria: (1) > 15% NLCD impervious coverage in the watershed, and (2) two-thirds of the years from 1985 to 2019 having < 10% missing streamflow data. For watersheds with nested streamflow gauges, we selected the one with the highest proportion of impervious cover, so that there were no overlapping watersheds. This filtering process resulted in a total of 219 watersheds with sizes from 1.6 to 1143 km^2^ distributed across the U.S. representing a wide range of hydroclimate conditions and geographic regions. For additional details on the watershed filtering process and USGS streamflow data processing, see Conley et al.^30^. Mean daily discharge data were downloaded from the USGS, quality checked, and processed using the R statistical programming software^65^ for the period 1985–2019. Because TELR models stormflow runoff, which includes surface and shallow subsurface flow, we separated baseflow from the USGS gauge data for validation comparison using the Hydrostats package in R^102^. Results of the watershed comparison are shown in Fig. 9, with an overall R^2^ value of 0.83 and percent bias of -7.1%. Best model performance was in smaller watersheds with > 30% impervious cover, with TELR showing moderate but consistent underprediction in watersheds with < 30% impervious cover.

### Estimating runoff changes and SCM performance

Total annual runoff change estimates for each study city were calculated from the 30-m gridded outputs from the TELR model, in which each grid cell represents the average annual runoff volume generated from that land area. The gridded outputs were generated for the historic period and the RCP 4.5 and 8.5 future scenarios. Runoff estimates for each city and scenario were quantified as the sum of the runoff output grid cells within the city's boundaries. We then calculated the percent difference in total annual runoff between the historic scenario and two future scenarios. City-scale runoff change raster maps were created by differencing the gridded runoff outputs, prior to summarizing by city boundaries.

To estimate SCM performance, a design standard representing the runoff depth generated from the historic scenario 85th percentile precipitation event was used as an input to the TELR runoff model. Although a variety of design standards are employed, the 24-h 85th percentile event standard is one of the most ubiquitous used across the United States^34^. To calculate untreated runoff for the historic scenario, all runoff generated from precipitation depths up to and including the historic 24-h 85th percentile depth was subtracted from the total runoff. To calculate untreated runoff for the two future precipitation scenarios, the same process was repeated with projected precipitation depths and the historic 85th percentile design standard. This approach assumes that all runoff generated up to the SCM design capacity is either infiltrated or treated, while flows above the design capacity are bypassed and left untreated. This approach also assumes that runoff is treated near to the source of generation as it is with distributed SCMs used for low impact development (LID), which often employ GSI. The final step was to calculate the percent difference in untreated runoff volume estimates for the historic and future scenarios. This difference represents the potential change in untreated runoff under future climate scenarios if SCM design standard depths remain static.

### Supplementary Information

Supplementary Information.

## Data Availability

The data used in this research and the scripts developed using R and the Google Earth Engine are available by contacting the authors.
