# Supplementary material for: Modeling the impact of future rainfall changes on the effectiveness of urban stormwater control measures
Source: Sci Rep. 2024 Feb 19;14:4082. doi: 10.1038/s41598-024-53611-1 (PMC10876621; doi:10.1038/s41598-024-53611-1)
Supplement: Supplementary file 1 — Supplementary Information. [file 41598_2024_53611_MOESM1_ESM.pdf]

Modeling the impact of future rainfall changes on the effectiveness of urban stormwater control measures

Tyler Nodine[1], Gary Conley[1], Catherine Riihimaki[1], Craig Holland[2] and Nicole Beck[1]

Supplementary Information

Table S1. CMIP5 global climate models (GCMs) used in this analysis. More details on each model are available at: pcmdi.llnl.gov/mips/cmip5/availability.html

Model Name	Developing Institution
ACCESS1-0	CSIRO (Commonwealth Scientific and Industrial Research Organisation, Australia), and BOM (Bureau of Meteorology, Australia)
bcc-csm1-1	Beijing Climate Center, China Meteorological Administration
BNU-ESM	College of Global Change and Earth System Science, Beijing Normal University
CanESM2	Canadian Centre for Climate Modelling and Analysis
CCSM4	National Center for Atmospheric Research
CESM1-BGC	National Science Foundation, Department of Energy, National Center for Atmospheric Research
CNRM-CM5	Centre National de Recherches Meteorologiques / Centre Europeen de Recherche et Formation Avancees en Calcul Scientifique
CSIRO-Mk3-6-0	Commonwealth Scientific and Industrial Research Organisation in collaboration with the Queensland Climate Change Centre of Excellence
GFDL-CM3	Geophysical Fluid Dynamics Laboratory
GFDL-ESM2G	Geophysical Fluid Dynamics Laboratory
GFDL-ESM2M	Geophysical Fluid Dynamics Laboratory
inmcm4	Institute for Numerical Mathematics
IPSL-CM5A-LR	Institut Pierre-Simon Laplace
IPSL-CM5A-MR	Institut Pierre-Simon Laplace
MIROC-ESM	Japan Agency for Marine-Earth Science and Technology, Atmosphere and Ocean Research Institute (The University of Tokyo), and National Institute for Environmental Studies
MIROC-ESM-CHEM	Japan Agency for Marine-Earth Science and Technology, Atmosphere and Ocean Research Institute (The University of Tokyo), and National Institute for Environmental Studies
MIROC5	Atmosphere and Ocean Research Institute (The University of Tokyo), National Institute for Environmental Studies, and Japan Agency for Marine-Earth Science and Technology
MPI-ESM-LR	Max Planck Institute for Meteorology (MPI-M)
MPI-ESM-MR	Max Planck Institute for Meteorology (MPI-M)
MRI-CGCM3	Meteorological Research Institute
NorESM1-M.	Norwegian Climate Centre

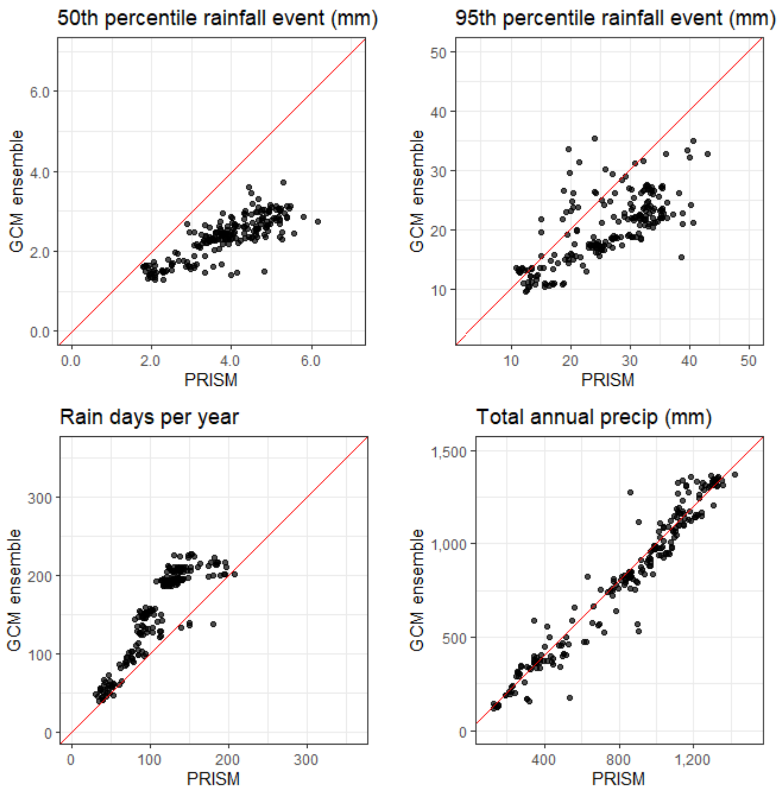

Figure S1. We evaluated performance of the GCM ensemble by comparing precipitation metrics calculated from the mean ensemble and those calculated from historic observed rainfall data from the PRISM Climate Group (Daly et al., 2008) for the period 1980-2005 (shown above). Rain days per year, the 50th percentile 24hr precipitation depth and the 95th percentile rainfall depth are three of the precipitation metrics used in the TELR runoff model (12.5^{th}, 85th, and 99th percentile depths are also used). Data points are individual raster pixels (4 km resolution) from both datasets intersecting the 23 study city boundaries.

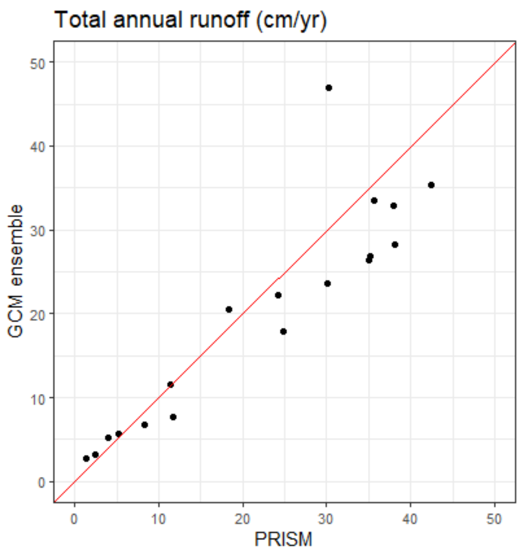

Figure S2. Comparison of total annual runoff modeled from PRISM precipitation data and the GCM ensemble for the period 1980-2005. Data points are the spatial mean of the total annual runoff depth for each study city (n=23). Runoff modeled from the two precipitation datasets showed strong alignment (R2=0.82) with a percent bias of -14.2% (runoff underestimation using the hindcasted GCM data).
